# Recruitment of Aβ into α‑Synuclein Condensates Catalyzes Primary Nucleation of α‑Synuclein Aggregation

**DOI:** 10.1021/acscentsci.5c00614

**Published:** 2025-07-28

**Authors:** Owen M. Morris, Alexander Röntgen, Zenon Toprakcioglu, Mariana Cali, Samuel Dada, Michele Vendruscolo

**Affiliations:** Centre for Misfolding Diseases, Yusuf Hamied Department of Chemistry, 2152 University of Cambridge, Cambridge CB2 1EW, U.K.

## Abstract

The aggregation of amyloid-β (Aβ) and α-synuclein (αSyn) is linked to Alzheimer’s and Parkinson’s diseases, with growing evidence suggesting possible interactions between Aβ and αSyn in the pathology of these neurodegenerative conditions. In this context, the recent observation that protein aggregation into amyloid fibrils may take place within liquid condensates generated through liquid–liquid phase separation prompts the question of how amyloidogenic proteins interact with each other, and more specifically whether Aβ can influence the overall phase behavior of αSyn or vice versa. To address this question, we investigated the interplay between Aβ40, the most abundant form of Aβ, with αSyn. We found that monomeric Aβ40 is sequestered into αSyn condensates, where it enhances heterogeneous primary nucleation, and accelerates the aggregation of αSyn within the liquid condensates. Using a chemical kinetics framework, we further showed that this liquid-to-solid transition is not significantly affected by adding preformed Aβ40 fibrillar seeds, further indicating that monomeric Aβ40 specifically enhances the primary nucleation of αSyn within the condensed phase. These findings identify some of the key mechanistic processes underlying amyloid aggregation within liquid condensates, prompting further investigations into the possible role of Aβ and αSyn cocondensation interactions in the onset and progression of neurodegenerative disorders.

## Graphical abstract

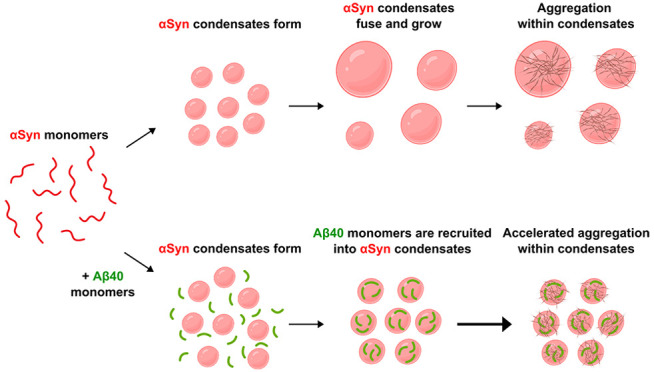

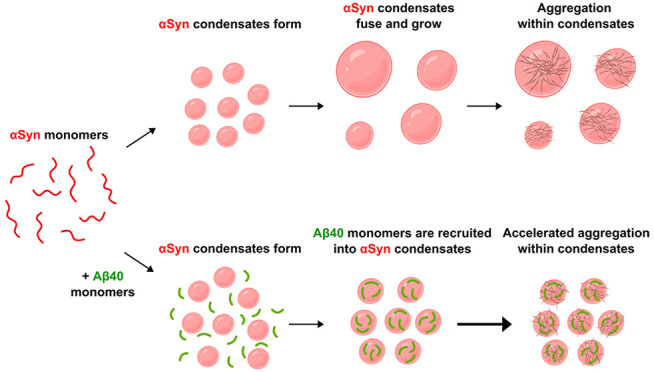

## Introduction

Neurodegenerative disorders, such as Alzheimer’s (AD) and Parkinson’s disease (PD), are associated with the misfolding and aggregation of proteins into the amyloid state.^1–5^ AD, the most prevalent cause of dementia, is linked with the deposition of the amyloid-β peptide (Αβ) into amyloid plaques,^1,2,6^ while PD, one of the most common movement disorders, is linked to the self-assembly of α-synuclein (αSyn) into Lewy pathology.^3,4,7,8^ Due to their association with disease, the aggregation processes of Aβ and αSyn have been extensively studied.^5,9–13^ Kinetic analyses have revealed that, initially, native proteins undergo primary nucleation to form a population of oligomeric nuclei.^12,14^ These nuclei grow further into amyloid fibrils by undergoing elongation.^10,12–15^ These amyloid fibrils can then catalyze the formation of new nuclei species by a process known as surface-catalyzed secondary nucleation.^5,9,10,14^

Since amyloid plaques have been observed to coexist with Lewy pathology in post-mortem samples from patients with PD and dementia with Lewy bodies (DLB),^16–22^ possible interactions between Aβ and αSyn are currently being studied.^23–27^ Increasing evidence suggests a potential mechanism whereby Aβ from the extracellular domain can be internalized into the cell via endocytic mechanisms.^28–31^ Once internalized and confined within the endosomal lumen, the concentration of Aβ in cell models may be enhanced by over 100-fold.^30^

A possible mechanism of the interaction between Aβ and αSyn may involve a new aspect of protein science, that of liquid–liquid phase separation.^32–34^ The assemblies formed by liquid–liquid phase separation are often referred to as biomolecular condensates.^32–35^ The incorporation of amyloidogenic proteins into condensed states creates a high local concentration and may potentially foster an environment that facilitates their nucleation.^9,32,36^ This process has been characterized for αSyn and Aβ, as well as other amyloidogenic proteins, such as FUS, tau, and TDP-43 on an individual protein basis.^37–46^

In this study, we investigate the interaction of Aβ with αSyn in the context of liquid–liquid phase separation. We find that adding Aβ40, the most abundant variant of Aβ, to αSyn increases the propensity of these liquid condensates to mature and age over time, resulting in their aggregation. We further show that the mechanism through which this occurs is that monomeric Aβ40 can be sequestered into αSyn condensates, which promotes heterogeneous primary nucleation. Through confocal microscopy, and a combination of biophysical and kinetic analysis, we demonstrate that monomeric Aβ40 disrupts the liquid-like character of these condensates in a concentration-dependent manner, thus affecting condensate number and size. Moreover, we show that by adding Aβ40 fibrils to the solution, αSyn condensate aggregation is minimally affected. This supports the idea that αSyn and Aβ40 monomeric interactions are potentially the driving force behind the accelerated liquid-to-solid transition that we observe.

## Results

### Aβ40 Is Recruited into αSyn Liquid–Liquid Phase Separated Condensates

A general workflow for investigating the recruitment of monomeric Aβ40 into αSyn condensates is shown in Figure 1. Following a previously established method,^38^ we deposited a small volume of the sample (see Methods) onto a microscope slide, and employed confocal microscopy to visualize the recruitment of monomeric Aβ40 into αSyn condensates that are formed through liquid–liquid phase separation.

**Figure 1:**
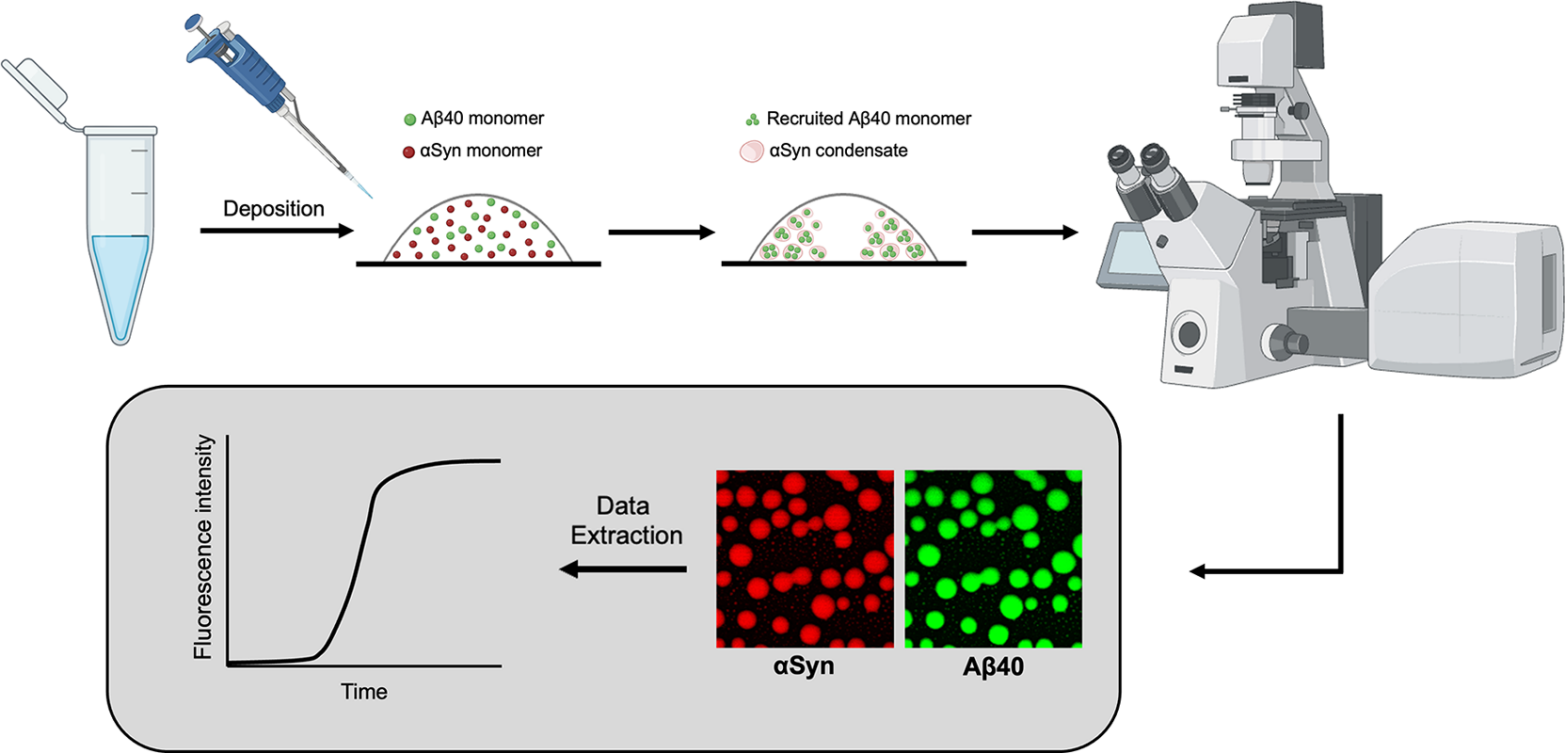
Schematic of the drop-casting assay used to monitor the liquid–liquid phase separation process of αSyn and Aβ40 and their aggregation within liquid condensates. A sample mixture was prepared comprising αSyn and Aβ40 (see Methods). A 10 μL sample was deposited onto a glass microscope dish, and confocal microscopy was utilized to monitor the emergence of αSyn condensates and the subsequent recruitment of Aβ40 within them. The aggregation within the condensed state was then characterized. Time-lapse images of the condensates were analyzed using Fiji.^75^ The depiction of the confocal microscope was adapted from BioRender.

To investigate the recruitment of Αβ40 into condensates formed of αSyn, we prepared samples containing a fixed concentration of αSyn (100 μM) with varying concentrations of monomeric Aβ40 (0.5–20 μM). For all experiments, triplicates were performed (n = 3). Moreover, to ensure that the results are reproducible and minimize any discrepancies, the experiments were conducted on different days, using different protein batches and on different microscope slides. To visualize the liquid–liquid phase separation process of αSyn and the subsequent recruitment of monomeric Aβ40, we fluorescently labeled both proteins (AF647-αSyn and AF488-Aβ40) (see Methods). To minimize and avoid potential influences on the phase separation processes from the dye, we used a 1%-labeled solution for αSyn and a 10%-labeled solution for Aβ40. The remainder of the solution consisted of 99% and 90%, unlabeled, wild-type protein, respectively for αSyn and Aβ40 (see Methods for more details). A small volume of each sample was pipetted onto a microscope dish, while confocal microscopy was utilized to monitor the liquid–liquid phase separation of the proteins. We observed coacervates formed via liquid–liquid phase separation for each condition tested (Figure 2, Supplementary Figure 1). The sample composed entirely of αSyn underwent liquid–liquid phase separation, and the resulting condensates exhibited characteristic liquid-like behavior through Ostwald ripening and coalescence, leading to the formation of the larger condensates observed at 10 min. On the other hand, the sample composed entirely of Aβ40 did not undergo liquid–liquid phase separation under these conditions (Supplementary Figure 2), thus indicating that αSyn is essential to driving the formation of condensates. Notably, as we introduced different concentrations of monomeric Aβ40 to the sample mixture, we observed the sequestration of monomeric Aβ40 into αSyn condensates (Figure 2). Furthermore, as we progressively increased the concentration of Aβ40, it was found that the condensates within the sample droplet appeared to be smaller in size and more numerous. These findings suggest that, as more monomeric Aβ40 is recruited into the condensates, the condensates undergo a liquid-to-solid transition, thereby preventing them from further fusing and coalescing, impairing the growth of the condensates. However, it should be noted that this effect could also be attributed to monomer depletion. That is to say that as the number of nuclei increases, the size of the nuclei decreases since there are fewer available monomers to promote nuclear growth.

**Figure 2:**
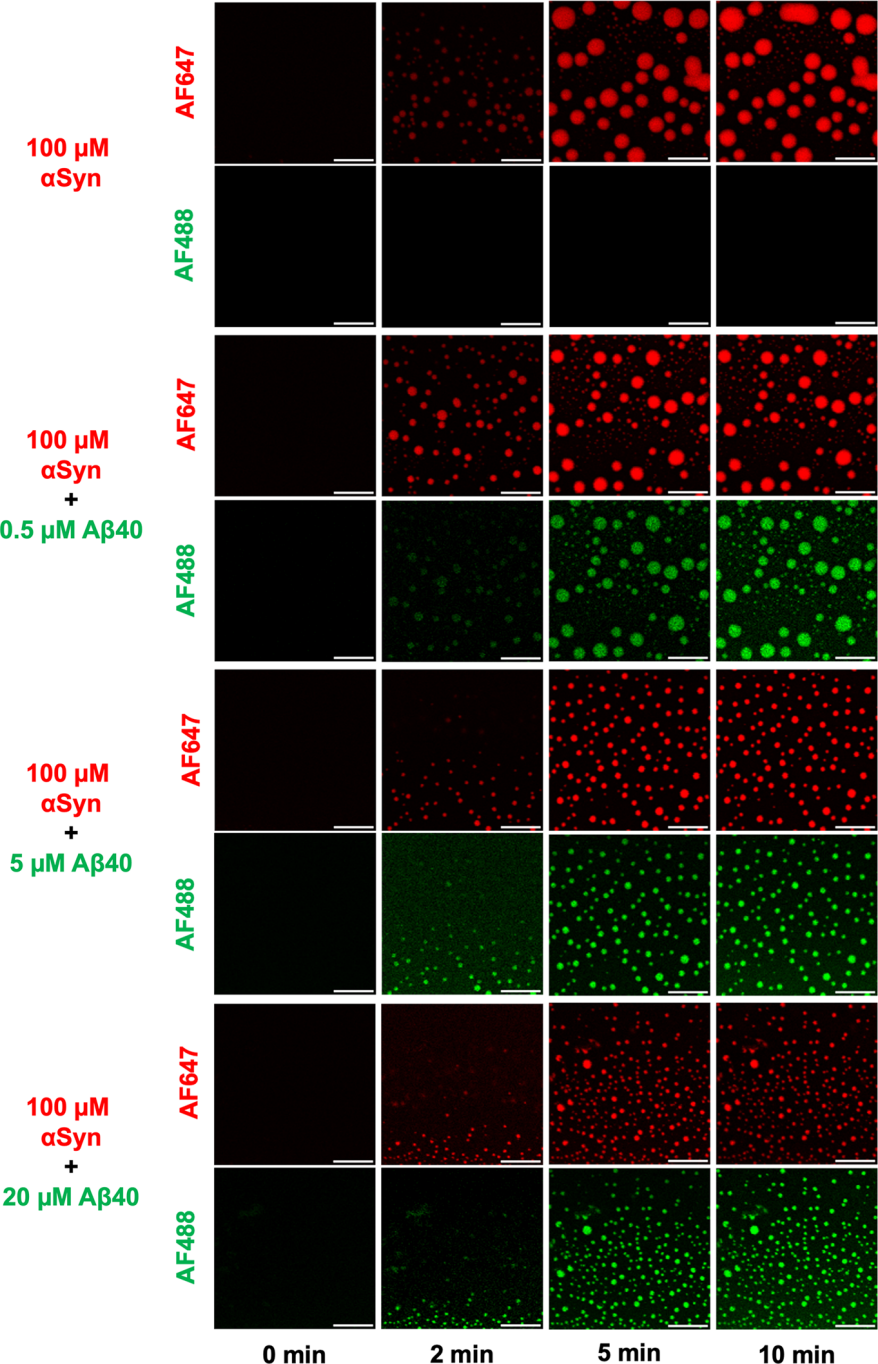
Recruitment of monomeric Aβ40 into αSyn condensates. Time-lapse confocal microscopy images displaying the liquid–liquid phase separation of αSyn (1% AF647-labeled) and the subsequent recruitment of increasing concentrations of monomeric Aβ40 (10% AF488-labeled, 0.5, 5, and 20 μM) into the liquid condensates. All experiments were conducted in triplicates (*n* = 3) and the images shown are representative of each condition tested.

### Kinetic Analysis Reveals That Recruitment of Monomeric Aβ40 into αSyn Condensates Promotes Heterogeneous Primary Nucleation

Given the notable decrease in the average area of αSyn condensates due to an increase in the concentration of monomeric Aβ40 recruited within condensates, we next sought to investigate the impact of increased monomeric Aβ40 sequestration and the effect this had on the aggregation propensity of these proteins. To study the aggregation of αSyn and Aβ40 within the condensates, we added 20 μΜ of the amyloid-binding dye thioflavin T (ThT) into the mixture. Once ThT is bound to the β-sheet core of amyloid fibrils, its quantum yield increases, resulting in an overall increase in fluorescent signal.^47,48^ Moreover, to minimize potential cross-talk between the emission intensity of AF488-labeled Aβ40 and ThT, which exhibits similar excitation/emission wavelengths to AF488, we only used wild-type Aβ40 to conduct these experiments.

Furthermore, we used confocal microscopy to investigate the incorporation of monomeric Aβ40 into αSyn condensates, and its effects on the overall protein aggregation within the condensed phase. Liquid–liquid phase separation was once again observed for all samples, followed by an increase in the ThT intensity (Figure 3). For the sample composed entirely of αSyn, ThT intensity was observed at approximately 8 min after the initiation of liquid–liquid phase separation. However, for all conditions tested where monomeric Aβ40 was added, the increase in ThT intensity was observed earlier, at around 5 min post liquid–liquid phase separation (Figure 3A). A negative control experiment was also conducted to determine whether Aβ40 by itself can phase separate. A sample composed entirely of Aβ40 was found to not undergo liquid–liquid phase separation, and subsequently, no ThT intensity was observed (Supplementary Figure 2A,B). This is in agreement with previous findings where only small molecules were able to induce and trigger Aβ40 liquid–liquid phase separation.^37^

**Figure 3:**
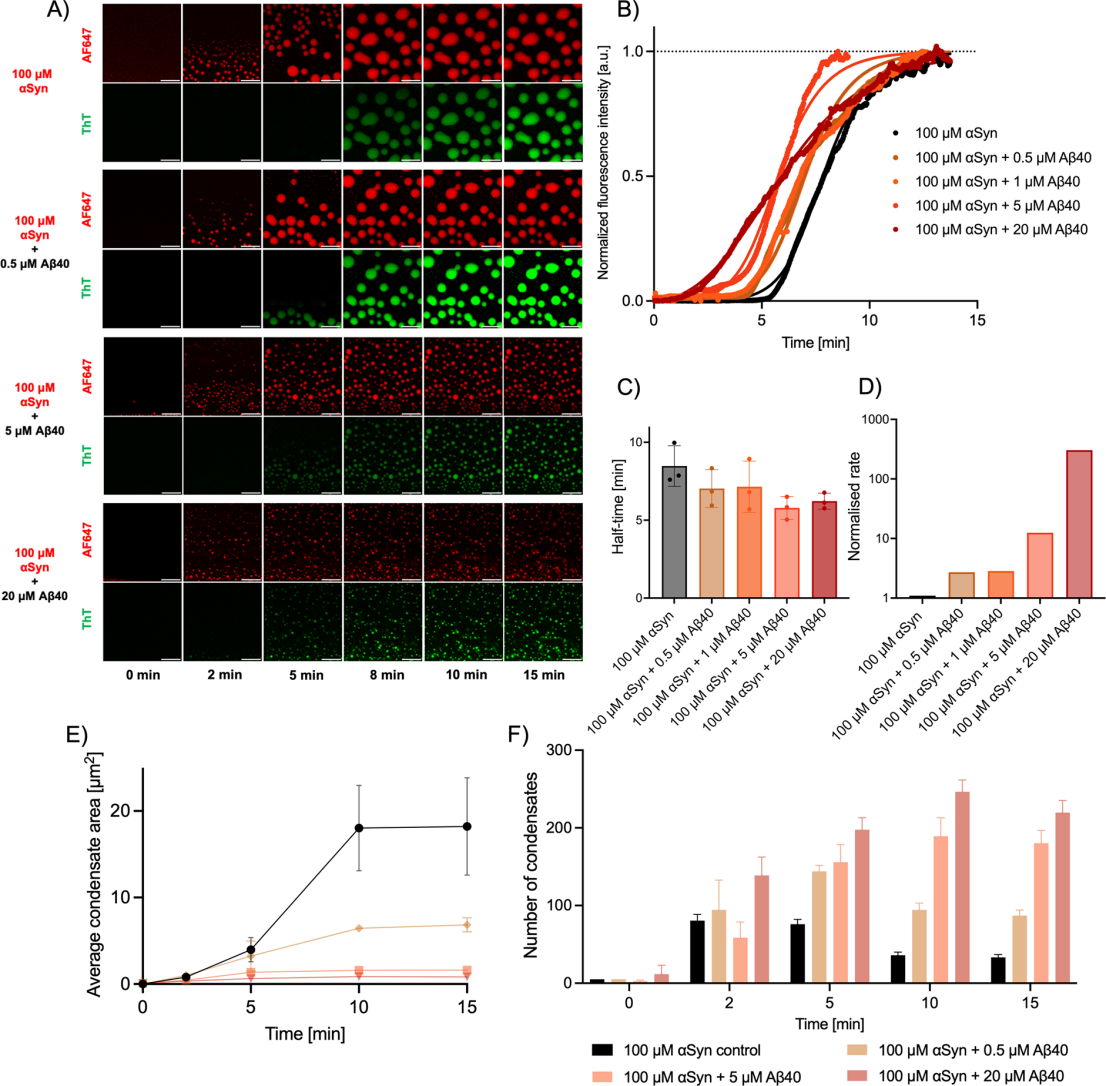
Monomeric Aβ40 enhances the aggregation of αSyn within liquid condensates. (A) Time-lapse confocal microscopy images of αSyn condensates with the addition of varying Aβ40 concentrations (AF647 channel). The ThT channel shows the aggregation process within the liquid condensates. Scale bar = 10 μm. (B) Normalized median kinetic traces corresponding to aggregation in the presence of increasing concentrations of monomeric Aβ40. Solid lines represent the fits from Amylofit.^49^ (C) Half-time of αSyn aggregation within liquid condensates with the addition of increasing concentrations of monomeric Aβ40. The statistical significance was analyzed by a one-way ANOVA test compared to αSyn control. The error bars are obtained from the deviation between replicates of different experimental trials. *n* = 3. (D) Graph of the aggregation rate as a function of increasing concentrations of monomeric Aβ40. (E) Decrease in the average area of αSyn condensates with the addition of increasing concentrations of monomeric Aβ40. *n* = 3. (F) Increase in the number of αSyn condensates over time upon addition of increasing concentrations of monomeric Aβ40. Analyzed condensates were selected based on those being in-focus within the confocal microscopy images. *n* = 3.

Our experiments yielded a series of ThT kinetic curves which were used to track the aggregation within the condensed state in the presence of various concentrations of monomeric Aβ40 (Figure 3B). The raw data of these kinetic curves are shown in Supplementary Figure 3A–E. These individual kinetic traces exhibited a characteristic sigmoidal pattern, with a rapid exponential growth phase of fibrils occurring after a relatively steady lag phase, followed by a plateau phase. Moreover, we found that increasing the concentration of monomeric Aβ40 in our sample mixture accelerated the aggregation within the condensed state. Furthermore, the degree of enhancement in the aggregation process within the condensed state was proportional to the increase in the concentration of Aβ40. This trend was corroborated by determining the half-time of aggregation, which is the time taken for half of the monomeric material to convert into fibrillar material. Here, as the concentration of Aβ40 incubated within the phase-separated sample is increased, the half-time of αSyn aggregation within the condensed state progressively decreases (Figure 3C).

To identify the microscopic processes that accelerate protein aggregation within the condensed phase upon the addition of monomeric Aβ40, we used Amylofit, a global fitting platform that compares the integrated rate laws to experimental data.^49^ These microscopic steps include primary nucleation (*k*_n_), elongation (*k*_+_), and surface-catalyzed secondary nucleation (*k*_2_). The reaction order of the primary process is denoted by *n*_c_, while the reaction order of the secondary process is denoted by *n*_2_. Despite the complexity of the kinetic network underlying the aggregation process, it has been reported that just two key rate parameters control the time course of aggregation.^14,50^ These are the combinations of rate constants *k*_+_ *k*_n_ and *k*_+_ *k*_2_, which describe aggregate proliferation through primary and secondary nucleation, respectively.^14,50^ By varying the *k*_+_ *k*_n_ rate constant, which contains the primary nucleation rate, and keeping the elongation and secondary nucleation rates *k*_+_ *k*_2_ constant, we were able to analyze our normalized kinetic data (Figure 3B). We found that the addition of monomeric Aβ40 accelerates the overall protein aggregation within the condensates through a mechanism consistent with heterogeneous primary nucleation. Typically, all nucleation events initiate either through homogeneous nucleation, or via heterogeneous nucleation. In the former case, protein molecules can spontaneously come together in the solution, resulting in nuclei formation. In the latter case, the protein molecules interact with surfaces, and in doing so they form nuclei at the interface of that surface. Additionally, it should be noted that due to the free energy, heterogeneous nucleation is more probable than homogeneous nucleation. The solid lines in Figure 3B represent the fits to the kinetic data. Remarkably, this mechanism of enhancing αSyn aggregation by Aβ-driven heterogeneous primary nucleation has been observed also in the *in vitro* deposition pathway.^27^ A kinetic analysis revealed that the combined rate is highly dependent on the concentration of monomeric Aβ40 within the system, and that for the highest concentration of monomeric Aβ40 tested, 20 μM, the rate is more than 2 orders of magnitude higher than the control, i.e. when αSyn is left to aggregate by itself through the condensed phase (Figure 3D).

To investigate the effect of this enhanced aggregation on the material properties of these condensates, we conducted an analysis of the size distribution of the condensates. The average condensate area was evaluated at various time points during the liquid–liquid phase separation process and subsequent condensate maturation. We found that the average condensate size becomes smaller as the concentration of Aβ40 is increased (Figure 3E). In addition, we measured the total number of condensates of the αSyn-Aβ40 condensates, finding that they become more numerous as the concentration of Aβ40 is increased (Figure 3F). These results are consistent with the recent report that the average size of the condensates is reduced when the concentration of monomeric protein decreases,^51^ which is observed here due to the accelerated formation of amyloid fibrils, leading to a decrease in the monomeric concentration of αSyn. Furthermore, a comparison of the maximum ThT intensity observed within condensates between samples composed of 100 μM αSyn and 100 μM αSyn + 20 μM Aβ40 monomer display a similar maximum intensity. This reinforces the idea that Aβ40 triggers the aggregation of αSyn within condensates and does not aggregate itself (Supplementary Figure 3F). This is further corroborated by the fact that when we left Aβ40 to phase separate and aggregate by itself (i.e., in the absence of αSyn), we not only observed no phase separation, but Aβ40 did not aggregate either. These results are summarized in Supplementary Figure 2. To investigate the morphology of the aggregates, we performed transmission electron microscopy (TEM). For all conditions investigated, elongated morphologies were identified, indicating that the protein aggregates had a characteristic fibrillar morphology (Figure 4).

**Figure 4:**
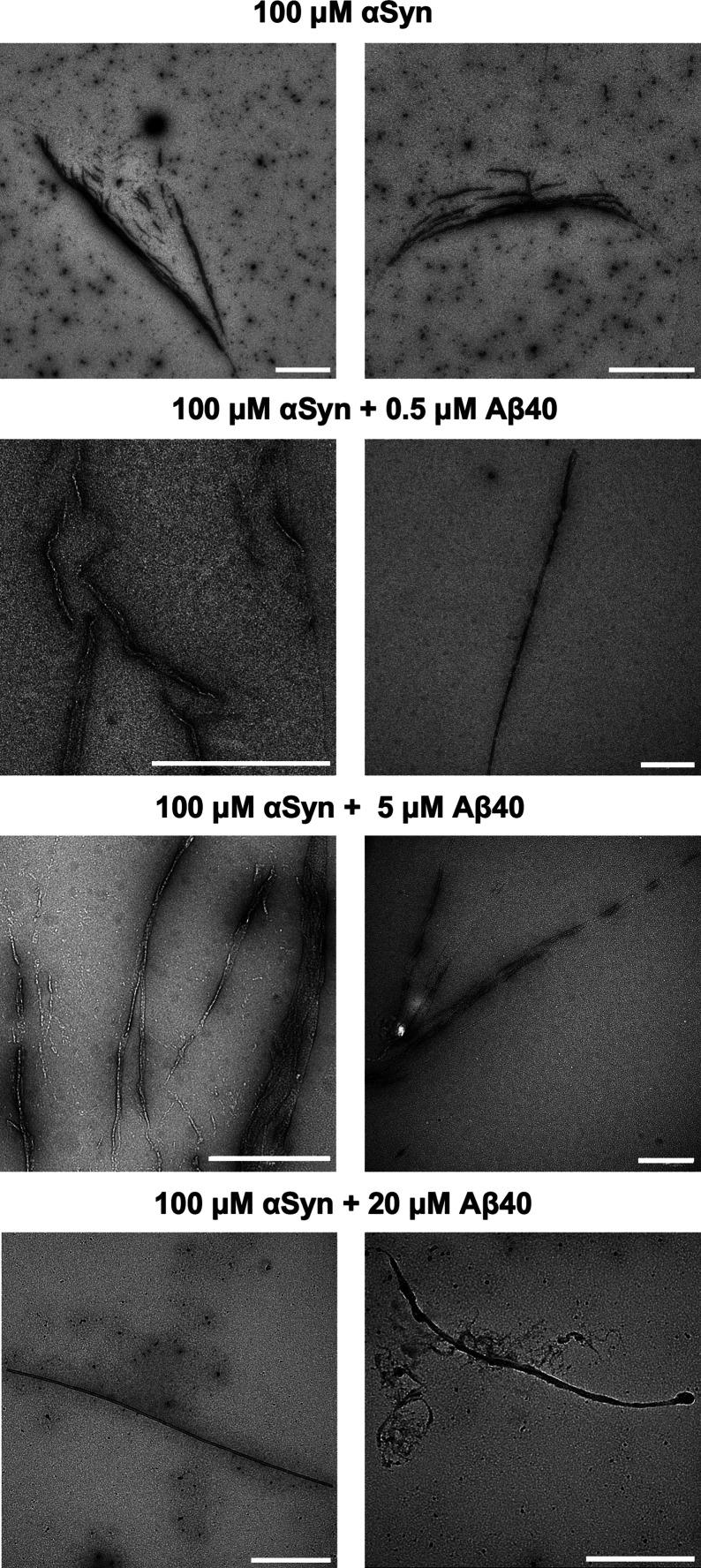
Transmission electron microscopy images of the fibrils generated within condensates. Fibrillar aggregates were imaged in the presence of various concentrations of Aβ40. Two micrographs are displayed for each condition. Scale bar = 1 μm.

### Kinetic Analysis Reveals That the Recruitment of Aβ40 Fibrillar Seeds into αSyn Condensates Slightly Promotes αSyn Aggregation

An important aspect of protein aggregation and condensation is the ability of preformed protein aggregates to template or “seed” these phase transition phenomena. To investigate whether the presence of Aβ40 fibrillar seeds could template αSyn aggregation via a heterogeneous secondary nucleation mechanism within the condensed state, we added various concentrations of Αβ40 fibrillar seeds into the phase-separated mixture. It was once again observed that for all conditions tested, αSyn underwent liquid–liquid phase separation in the presence of Αβ40 (Figure 5A), followed by subsequent aggregation within the condensates. The experimental kinetic traces indicate that the presence of Aβ40 fibrillar seeds accelerates the aggregation process within the condensed state compared to the control sample, which was comprised of an entirely monomeric sample of αSyn. However, there were no discernible concentration-dependent trends observed in terms of the efficacy of these Aβ40 fibrillar seeds in propagating αSyn fibril formation through a heterogeneous secondary nucleation mechanism (Figure 5B, C). While this implies that heterogeneous secondary nucleation plays a role, it is not the dominant pathway through which this system phase separates and aggregates.

**Figure 5:**
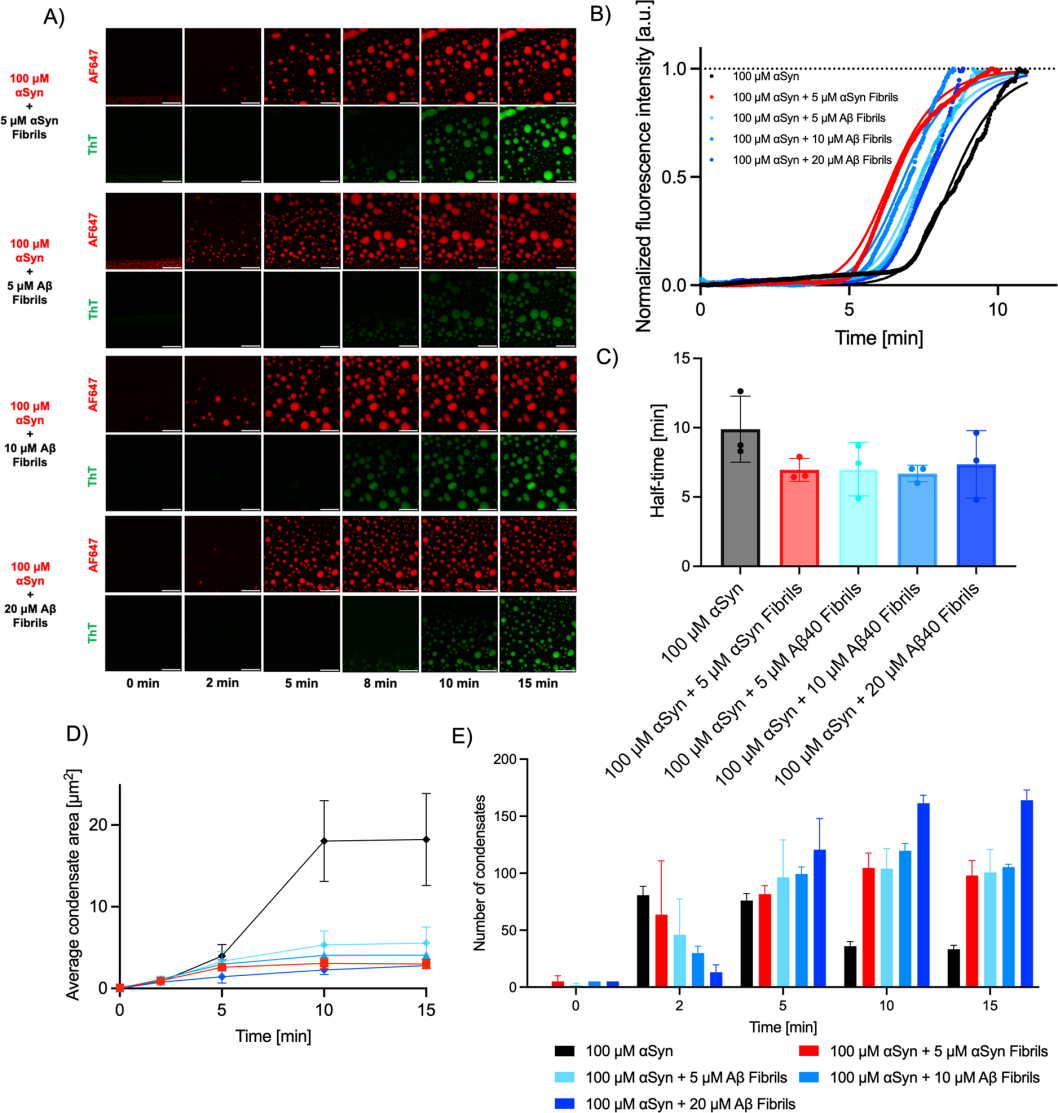
Recruitment of preformed Aβ40 fibrils into αSyn condensates. (A) Time-lapse confocal microscopy images of αSyn condensates with the addition of various concentrations of preformed αSyn and Aβ40 fibrillar seeds (AF647 channel). The ThT channel shows the subsequent protein aggregation within the condensed phase. Scale bar = 10 μm. (B) Normalized median kinetic traces corresponding to aggregation in the presence of an increasing concentration gradient of preformed fibrillar seeds. Solid lines represent the fits from Amylofit.^49^ (C) Half-time of aggregation within liquid condensates with the addition of varying concentrations of preformed fibrillar seeds, denoted as seeds. *n* = 3. (D) The recruitment of preformed Αβ40 fibrillar seeds has an effect on the average area of the condensates. *n* = 3. (E) Increasing concentrations of preformed Aβ40 fibrillar seeds, denoted as seeds, has an impact on the number of observed condensates. Analyzed condensates were selected based on those being in-focus within the selected confocal microscopy images. *n* = 3.

We further explored this through a chemical kinetics framework. We performed a similar kinetic analysis for the seeded data, again using Amylofit.^49^ To conduct the analysis, the concentration of αSyn within the condensate was used. To perform the fitting analysis, the addition of heterogeneous fibrillar seeds, i.e., Αβ40 fibrillar seeds added to αSyn, was treated as if they were external molecules. The addition of increasing concentrations of Αβ40 fibrillar seeds to the mixture did not incrementally accelerate the αSyn condensate liquid-to-solid transition (Supplementary Figure 4).

Furthermore, there was a reduction in the average condensate size when Aβ40 seeds were added to the solution. However, it did not vary significantly when exposed to increasing concentrations of 5–20 μM of Aβ40 fibrillar seeds (Figure 5D). Moreover, we looked at the number of condensates as a function of time. As expected, the αSyn alone followed a similar profile to that reported earlier (Figure 3) and the number of condensates reduced over time. On the other hand, when Aβ40 fibrillar seeds were added, we observed an inverse relationship in that the condensate number initially increased and then plateaued without decreasing. We believe that this can be attributed to the condensates having a reduced mobility when the Aβ40 fibrils are added, thus directly affecting their propensity of fuse (Figure 5E).

## Discussion

In light of the increasing evidence suggesting that the interaction between Aβ and αSyn is directly implicated in a plethora of neurodegenerative disorders, including Alzheimer’s and Parkinson’s diseases, it is crucial to better understand how these proteins influence one another. Only in this manner will we be able to fully unravel the mechanisms behind protein aggregation and dementia. It has been previously reported that Aβ can interact with αSyn at various stages of the aggregation process.^52^ In line with these observations, multiple studies have investigated the residues involved in the interaction, suggesting that the hydrophobic C-terminal residues as well as the central region of Aβ may play a critical role in binding αSyn.^23,24,52–54^

In this study, we build on and further expand previous work by specifically exploring how these proteins affect each other during liquid–liquid phase separation. In particular, we investigate how Aβ40 influences the liquid–liquid phase separation of αSyn in the condensation pathway. Through a combination of confocal microscopy, biophysical assays, electron microscopy, and kinetic analysis, we find that monomeric Aβ40 is recruited into αSyn condensates. More importantly, we have found that this process promotes the aggregation of αSyn within condensates via heterogeneous nucleation. A schematic representation of the proposed pathway by which monomeric Aβ40 is recruited into αSyn condensates, resulting in the enhancement, or acceleration, of the aggregation process, is shown in Figure 6. By performing kinetic analysis, we found an enhancement in the primary nucleation rate of αSyn condensate aggregation when increasing amounts of monomeric Aβ40 are added to the system. Furthermore, the analysis could be complemented with centrifugation methods and measurements of fluorescence intensity of fluorophore-labeled Aβ40 to determine the exact amount of Aβ40 monomers that have entered αSyn condensates.^39,55^ It does appear, however, that based on the fluorescent micrographs, most of Aβ40, if not all, is recruited into αSyn condensates. Moreover, we observed that adding Aβ40 to the system significantly stalled condensate growth, resulting in smaller condensates. This finding implies that Aβ40 monomers enhance the liquid-to-solid transition of αSyn. This process could be independently observed by fluorescence recovery after photobleaching (FRAP), which is in line with the increase in ThT intensity as a measurement of amyloid formation.^39^

**Figure 6:**
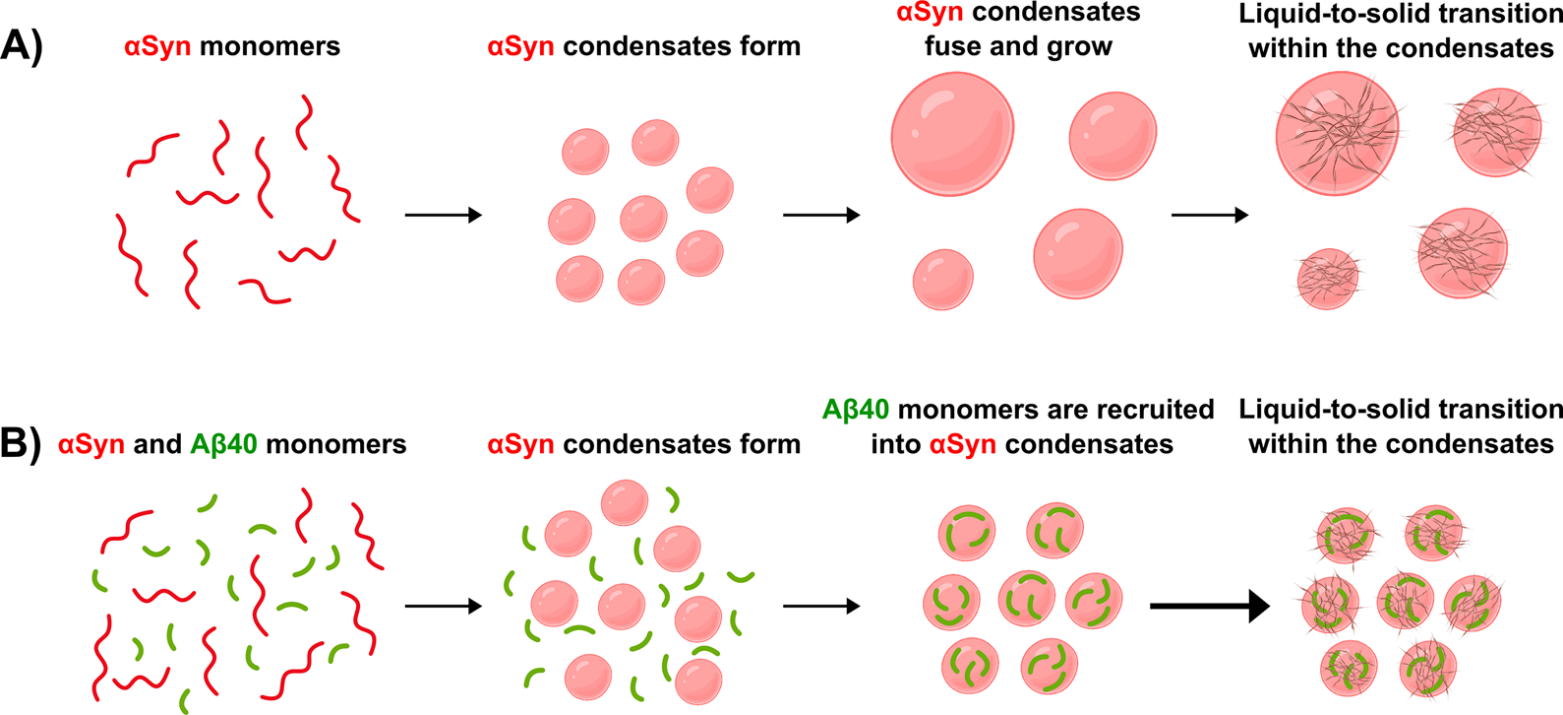
The recruitment of monomeric Αβ40 (green) into αSyn (red) condensates accelerates the phase transition process into amyloid aggregates. The results that we reported in this work indicate that Aβ40 monomers are recruited into αSyn condensates, where they accelerate the αSyn aggregation process. (A) αSyn in the absence of Aβ40 forms condensates that fuse and grow over time. Eventually, αSyn inside the condensates aggregates into amyloid fibrils. (B) When mixing αSyn with Aβ40, αSyn first forms condensates into which Aβ40 monomers are recruited. Within the condensates, Aβ40 then promotes the primary nucleation of αSyn and thereby accelerates the aggregation into amyloid fibrils. Moreover, the faster liquid-to-solid transition of αSyn in the presence of Aβ40 leads to a stalling of condensate growth, resulting in overall smaller and more numerous condensates compared to the control condition without Aβ40.

In contrast, we found that adding preformed Aβ40 fibril seeds had no significant effect in modulating the aggregation of αSyn within condensates. Moreover, it should be noted that oligomers of Aβ, which represent an intermediate species between monomers and amyloid fibrils, have been widely implicated in AD pathology as being potently neurotoxic and compromising synaptic function.^56,57^ Given their different conformations compared to disordered monomers and highly structured fibrils of Aβ, oligomers may thus exert distinct effects by modulating the surface dynamics and structural organization of αSyn condensates.^57^ Further work elucidating the effects of Aβ oligomers on αSyn condensation will thus be crucial to fully understand the interplay of both proteins.

Additionally, while Aβ40 is the most abundant Aβ species in the human brain, other variants such as Aβ42 are significantly involved in the insoluble plaque formation found in the brain parenchyma of AD patients.^58,59^ Moreover, Aβ42 is well-known to have a strongly enhanced aggregation propensity relative to Aβ40 due to its two additional hydrophobic, C-terminal amino acids, isoleucine and alanine.^60^ Similarly, we have recently shown that sequence variations arising from alternative splicing greatly affect the phase behavior of αSyn,^41^ and there is increasing evidence that these splice isoforms play an important role in the pathogenesis of synucleinopathies.^41,61,62^ The comparison of different proteoforms of both Aβ and αSyn would therefore greatly expand our understanding of the sequence-encoded, biophysical rules which govern the co-condensation of the two proteins. In fact, by considering the intricate behavior between protein variants, like those discussed above, one could understand the complex relationship between co-condensation and coaggregation in the context of neurodegenerative disorders.

In a broader context, mounting evidence suggests that the observation of comorbidities in neurodegeneration is not limited merely to Aβ and αSyn, but extends to many other amyloidogenic proteins such as tau, TDP-43 and prion protein.^19,63,64^ Co-pathologies of these proteins occur in multiple combinations and to varying degrees in the brain, creating a wide spectrum of interrelated, partially overlapping neurodegenerative conditions rather than clearly distinct disorders. Of note, up to date, several of the above-mentioned proteins have been shown to undergo co-condensation together via a liquid–liquid phase separation pathway.^65–67^ Furthermore, these findings are in line with reports showing that amyloidogenic proteins are capable of interacting with a variety of proteins and peptides during condensation and aggregate formation.^68–71^ It is therefore plausible that liquid–liquid phase separation represents a viable avenue for the interaction and coaggregation of amyloidogenic proteins. Disentangling the effect that each of these proteins exerts on one another will thus be central to dissecting their individual roles in amyloid formation and disease.

In conclusion, to understand the etiology of AD, synucleinopathies and other neurodegenerative disorders, it is critical to elucidate the molecular interactions driving disease progression. The results presented in this study offer insights into the complex relationship of αSyn and Aβ through liquid–liquid phase separation, which represents an efficient pathway through which these proteins can aggregate. Our finding that Aβ influences αSyn phase separation and aggregation contributes to our current understanding of amyloidogenic protein co-condensation. This work is thus part of a wider framework in the emerging concept of protein copathologies, which is likely to be essential for the successful development of effective therapeutic strategies to combat neurodegeneration in the future.

## Materials and Methods

### Purification and Labeling of αSyn

We expressed human wild-type αSyn and its cysteine-bearing variant (A90C) in *E. coli* (BL21 Gold (DE3) competent cells), which were transformed with a pT7-7 plasmid encoding the protein constructs. We then purified αSyn in 50 mM trisaminomethane-hydrochloride (Tris-HCl) at pH 7.4 following previously reported protocols.^62,72^ We labeled the A90C αSyn variant with an excess of Alexa Fluor 647 C_5_ maleimide (AF647, Invitrogen Life Technologies) overnight at 4 °C with continuous inversion. We then removed any unbound dye using Amicon Ultra-15 Centrifugal Filter Units and performed a buffer exchange into 50 mM Tris-HCl, pH 7.4. We determined the final labeled protein concentration from the Beer–Lambert law by measuring the UV/vis absorbance at 650 nm using the extinction coefficient ε = 239,000 M^–1^ cm^–1^ for AF647.

### Purification and Labeling of Aβ40

We expressed human wild-type Aβ40 and its cysteine-bearing variants (cysteine insertion at position 2) *E. coli* (BL21 Gold (DE3) competent cells), which were transformed with a pT7 plasmid encoding each Aβ variant in turn. We subsequently purified the Aβ variants in 50 mM Tris-HCl, pH 7.4, using previously reported protocols.^73,74^ All Aβ was aliquoted, flash-frozen in liquid nitrogen, lyophilized, and stored at −80 °C. We redissolved lyophilized Aβ in 50 mM Tris-HCl, pH 7.4, on ice at a stock concentration of (50 μM) before each experiment. We independently redissolved the cysteine-bearing Aβ40 and Aβ40 variants in 50 mM sodium phosphate buffer, pH 7.5, labeled with an excess of Alexa Fluor 488 C_5_ maleimide (AF488, Invitrogen Life Technologies) and incubated them at room temperature for 2 h. We separated labeled Aβ from unbound dye and Aβ dimers by size exclusion chromatography (Superdex 75 10/300 GL column) using a flow rate of 0.7 mL/min 50 mM Tris-HCl, pH 7.4. We determined the concentration of labeled Aβ from the Beer–Lambert law by measuring the UV/vis absorbance at 495 nm using the extinction coefficient ε = 73,000 M^–1^ cm^–1^ for AF488.

### Liquid–Liquid Phase Separation Assay

We took the liquid–liquid phase separation assay adopted in this work from a previously reported protocol.^39^ In brief, we prepared a sample mixture in 50 mM Tris-HCl, pH 7.4, consisting of 100 μM αSyn (1% AF647-labeled), 0.5–20 μM Aβ40 (10% AF488-labeled), and 5% (w/w) polyethylene glycol (PEG). We pipetted 10 μL of the sample using a drop-casting approach onto glass-bottom dish. We immediately imaged the sample using confocal microscopy (Leica Stellaris 5), setting the excitation wavelengths at 650 nm for αSyn (AF647) and 490 nm for Αβ40 (AF488). All images were processed and analyzed using Fiji.^75^

### Protein Aggregation Assay within Liquid Condensates

To study the aggregation of proteins within condensates, the sample mixture outlined above was supplemented with 20 μM thioflavin T (ThT). Only wild-type Αβ40 was used to eliminate potential overlaps between the fluorescently labeled protein and ThT. Ten μL of the sample was drop-casted onto a glass-bottom dish and was imaged using confocal microscopy (Leica Stellaris 5) at excitation wavelengths 650 nm αSyn and 405 nm for ThT. The ThT fluorescence intensity was extracted from time-lapse images analyzed using Fiji.^75^

### Generation of Fibril Seeds

We generated preformed fibrils of αSyn and Aβ40 by incubating each respective monomeric protein at 37 °C until a maximum fibrillar mass was achieved. We determined the concentration of αSyn and Aβ40 fibrils by UV/vis spectroscopy.

### Seeded Aggregation Assay within Liquid Condensates

We supplemented 100 μΜ αSyn (1% AF647-labeled) with either 5 μM αSyn fibrillar seeds or 5–20 μM of Aβ40 fibrillar seeds and 5% (w/w) PEG-10000 in 50 mM Tris-HCl, pH 7.4. Prior to use, we homogenized fibrillar seeds of αSyn and Aβ40 by sonication. We drop-casted 10 μL of this sample mixture onto a glass-bottom dish and imaged it using confocal microscopy (Leica Stellaris 5) at excitation wavelengths 650 nm αSyn and 405 nm for ThT. We extracted the ThT fluorescence intensity from time-lapse images analyzed using Fiji.^75^

### Transmission Electron Microscopy (TEM)

We performed glow-discharging of TEM grids (continuous carbon film on 300-mesh copper grid) using Quorum Technologies GloQube instrument at a current of 25 mA for 60 s. We spotted 5 μL of samples on grids for 5 min, washed them three times with 5 μL ultrapure water for 30 s, negatively stained them with 5 μL 2% (w/v) uranyl acetate solution for 40 s and finally air-dried them. We then performed TEM imaging using a Talos F200X G2 electron microscope.

### Statistical Analysis

We performed all statistical analyses in GraphPad Prism 9 (GraphPad Software). We present all data as the mean from at least 3 independent biological replicates, unless indicated otherwise.

## Supplementary Material



## Notes

Additional figures including mixtures of αSyn with Aβ40 as well as Aβ40 monomer-only control (PDF)
