# Supplementary material for: Recruitment of Aβ into α‑Synuclein Condensates Catalyzes Primary Nucleation of α‑Synuclein Aggregation
Source: ACS Cent Sci. 2025 Jul 28;11(8):1481–91. doi: 10.1021/acscentsci.5c00614 (PMC12395293; doi:10.1021/acscentsci.5c00614)
Supplement: Supplementary file 1 [file oc5c00614_si_001.pdf]

SUPPORTING INFORMATION

Recruitment of Aβ into α-Synuclein Condensates Catalyzes Primary Nucleation of α-Synuclein Aggregation

Owen M. Morris[1,+], Alexander Röntgen[1,+], Zenon Toprakcioglu[1,+*],
Mariana Cali[1], Samuel Dada[1], and Michele Vendruscolo[1*]

[1]*Centre for Misfolding Diseases, Yusuf Hamied Department of Chemistry, University of Cambridge, Cambridge CB2 1EW, UK*

+ *Equal contributions*
* *Correspondence to: zt231@cam.ac.uk, mv245@cam.ac.uk*

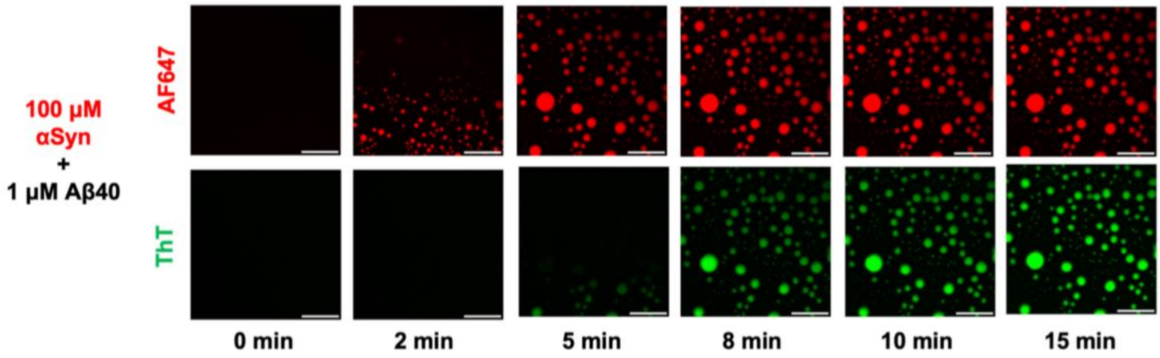

Figure S1. Time-lapse confocal microscopy images displaying αSyn condensates with the addition of monomeric Aβ40 concentrations. ThT channel shows the subsequent protein aggregation within the condensed phase. Scale bar = 10 μm.

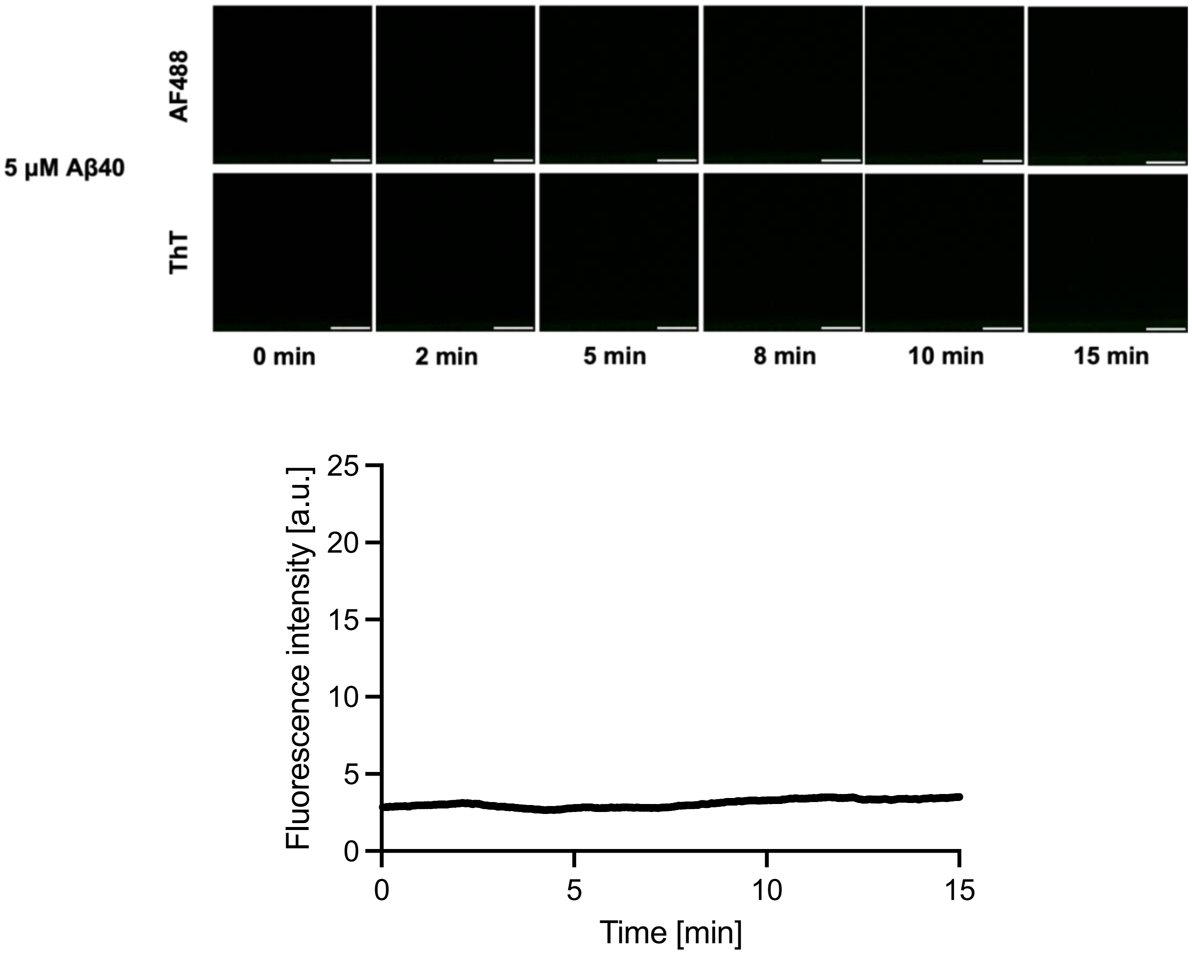

Figure S2. Negative control for the liquid-liquid phase separation of Aβ40 in the absence of αSyn. (A) Time-lapse confocal microscopy images of 5 μM Aβ40 with 5% (w/w) PEG. Under these conditions, in the absence of αSyn, Aβ40 does not undergo liquid-liquid phase separation. ThT channel indicates that Aβ40 does not undergo aggregation via a liquid-condensation pathway. Scale bar = 10 μm. **(B)** ThT kinetic aggregation data indicating that Aβ40 aggregation is not detectable when a 10 μL sample droplet is suspended onto a microscope slide. Data are shown as mean of n=3.

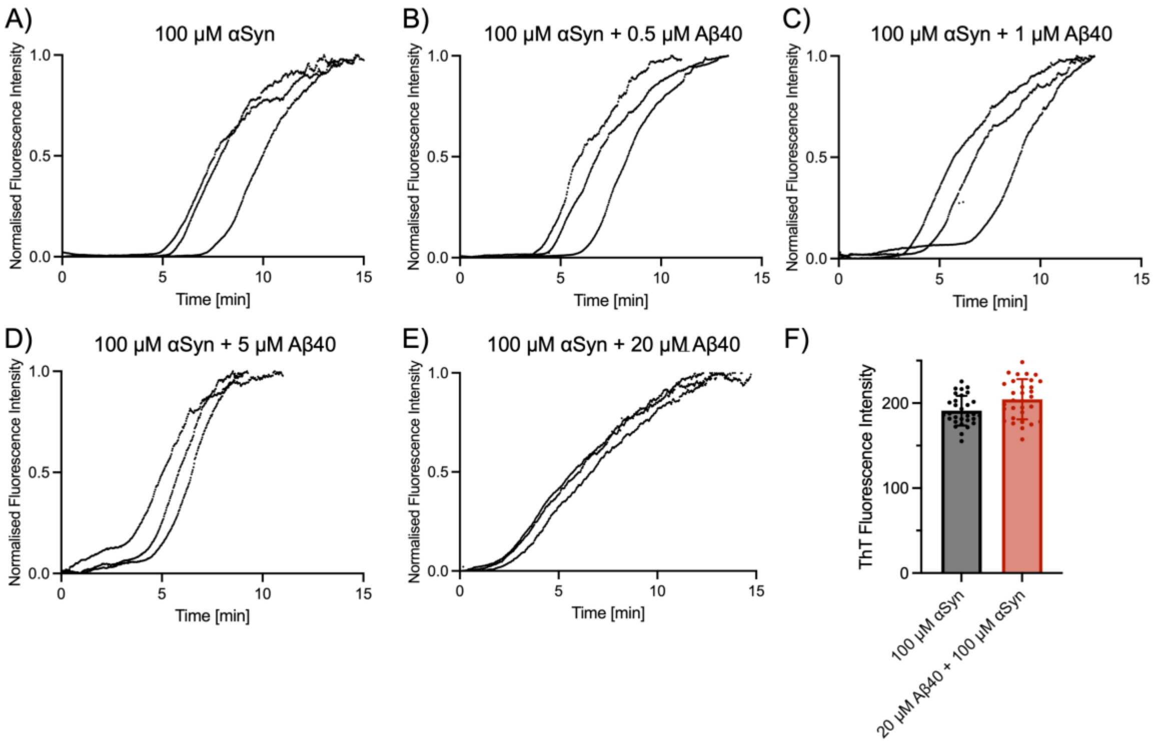

Figure S3. Increasing concentrations of monomeric Aβ40 accelerates the aggregation of αSyn in condensates. (A–E) Raw kinetic traces for αSyn aggregation via the condensation pathway in the presence of various concentrations of Aβ40. **(F)** Maximum ThT intensity of αSyn condensates for samples composed of 100 μM αSyn vs. 100 μM αSyn + 20 μM Aβ40, n=30.

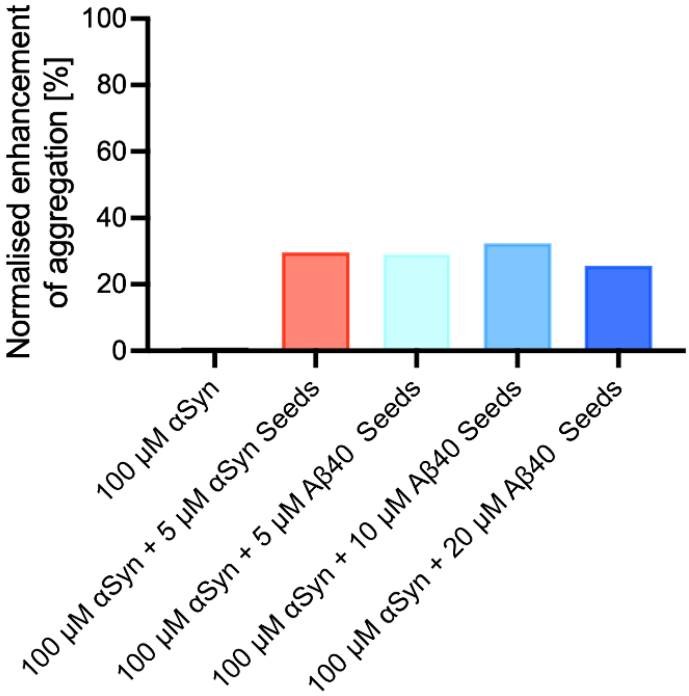

Figure S4. Bar chart comparing the enhancement of protein aggregation as fibrillar seeds are introduced to condensates. The enhancement is calculated by the percentage increase in the half-time of protein aggregation, relative to the 100 μM αSyn control.
